# Long non-coding RNA-associated competing endogenous RNA axes in the olfactory epithelium in schizophrenia: a bioinformatics analysis

**DOI:** 10.1038/s41598-021-04326-0

**Published:** 2021-12-30

**Authors:** Hani Sabaie, Marziyeh Mazaheri Moghaddam, Madiheh Mazaheri Moghaddam, Nazanin Amirinejad, Mohammad Reza Asadi, Yousef Daneshmandpour, Bashdar Mahmud Hussen, Mohammad Taheri, Maryam Rezazadeh

**Affiliations:** 1 Student Research Committee, Tabriz University of Medical Sciences, Tabriz, Iran; 2 Department of Medical Genetics, Faculty of Medicine, Tabriz University of Medical Sciences, Tabriz, Iran; 3 Department of Genetics and Molecular Medicine, School of Medicine, Zanjan University of Medical Sciences (ZUMS), Zanjan, Iran; 4 Department of Biology, Faculty of Sciences, Shahid Bahonar University of Kerman, Kerman, Iran; 5 Department of Pharmacognosy, College of Pharmacy, Hawler Medical University, Erbil, Kurdistan Region Iraq; 6 Men’s Health and Reproductive Health Research Center, Shahid Beheshti University of Medical Sciences, Tehran, Iran; 7 Clinical Research Development Unit of Tabriz Valiasr Hospital, Tabriz University of Medical Sciences, Tabriz, Iran

**Keywords:** Genetics, Neuroscience, Physiology

## Abstract

The etiology of schizophrenia (SCZ), as a serious mental illness, is unknown. The significance of genetics in SCZ pathophysiology is yet unknown, and newly identified mechanisms involved in the regulation of gene transcription may be helpful in determining how these changes affect SCZ development and progression. In the current work, we used a bioinformatics approach to describe the role of long non-coding RNA (lncRNA)-associated competing endogenous RNAs (ceRNAs) in the olfactory epithelium (OE) samples in order to better understand the molecular regulatory processes implicated in SCZ disorders in living individuals. The Gene Expression Omnibus database was used to obtain the OE microarray dataset (GSE73129) from SCZ sufferers and control subjects, which contained information about both lncRNAs and mRNAs. The limma package of R software was used to identify the differentially expressed lncRNAs (DElncRNAs) and mRNAs (DEmRNAs). RNA interaction pairs were discovered using the Human MicroRNA Disease Database, DIANA-LncBase, and miRTarBase databases. In this study, the Pearson correlation coefficient was utilized to find positive correlations between DEmRNAs and DElncRNAs in the ceRNA network. Eventually, lncRNA-associated ceRNA axes were developed based on co-expression relations and DElncRNA-miRNA-DEmRNA interactions. This work found six potential DElncRNA-miRNA-DEmRNA loops in SCZ pathogenesis, including, *SNTG2-AS1*/*hsa-miR-7-5p*/*SLC7A5*, *FLG-AS1*/*hsa-miR-34a-5p*/*FOSL1*, *LINC00960*/*hsa-miR-34a-5p*/*FOSL1*, *AQP4-AS1*/*hsa-miR-335-5p*/*FMN2*, *SOX2-OT*/*hsa-miR-24-3p*/*NOS3*, and *CASC2*/*hsa-miR-24-3p*/*NOS3*. According to the findings, ceRNAs in OE might be promising research targets for studying SCZ molecular mechanisms. This could be a great opportunity to examine different aspects of neurodevelopment that may have been hampered early in SCZ patients.

## Introduction

A century after an influential work done by Kraepelin on the subject of dementia praecox, schizophrenia spectrum disorder (SSD) is still mysterious in terms of its different etiologies, symptomatology, the unpredictability of disease progression, moderate therapeutic outcomes, and the concern of comorbidities like obesity, diabetes, and tobacco use disorder^1–3^. The term SSD refers to a wide variety of symptoms, and neither all SSD patients express the entire symptoms, nor all of the corresponding symptoms occur at the same time. SSD includes schizophrenia (SCZ), schizophreniform disorder, schizoaffective disorder, and schizotypal personality disorder^4^. Patients with SSD usually display disorganization in formal thinking and language, catatonic symptoms, hallucinations, delusions, affect and mood dysfunctions, self-disorder, neurocognitive deficits, and somatic symptoms. Nearly a half of SSD patients have functional impairments, which raise the likelihood of constant unemployment and the difficulty to form and sustain lasting relationships^2,3^. Neuronal, psychological, social, environmental, and genetic variables might lead to SSD formation and maintenance^3,5,6^. Modifications in the morphology of neurons and the brain are thought to be linked to SSD symptomatology^5,6^. Furthermore, studies show that being at younger ages at the onset of disease, experiencing suicide attempts, having a progressive disease onset, and facing difficulty adhering to treatment are all risk factors for recurrence in SSD people^3^. Based on animal studies and imaging data obtained from the patients, it is improbable that schizophrenia is caused by a traditional degenerative process^2^. Defective oligodendrocyte functions, synaptogenesis, and perhaps decreased neurogenesis, with accompanying deficiencies in structural and functional micro- and macroconnectivity, indicate a disruption in the human brain's regenerative potential in schizophrenia^2^. As regards possible underlying neurophysiological and genetic factors, it appears possible that SDD may be considered a failed neuro-regeneration^2^. Aberrant gene expression and protein production are associated with SCZ pathophysiology, and these alterations in SCZ patients occur in multiple brain regions and have temporal variation during disease progression^7–9^. More importantly, a growing body of evidence has indicated alterations in non-coding RNAs (ncRNAs) in SCZ patients^10^. These findings help elucidate the molecular mechanisms underlying the dysregulation of gene expression and protein production. The ncRNAs comprise various classes of RNA transcripts with different lengths^11^. Accumulating evidence has revealed the aberrant expression of microRNAs (miRNAs) (20–22 nucleotides)^12,13^ and long non-coding RNAs (lncRNAs), with over 200 nucleotides, in the brain of SCZ patients, which implicates in the occurrence and development of SCZ^14,15^. Pandolfi et al. in 2011 proposed competing endogenous RNA (ceRNA) theory as a new regulatory mechanism. They suggested that cross-talk between coding and ncRNAs (including lncRNAs, circular RNAs (circRNAs), and pseudogenes) forms a massive regulatory network across the diverse components of the transcriptome through the miRNA response elements. The ceRNA theory posits that the expression level of two RNA transcripts inversely correlates with target miRNAs levels. Moreover, the expression levels of these two RNA transcripts correlate positively with each other^16^. Many studies have corroborated the ceRNA theory, as emerging evidence verified that ceRNA cross-talk imbalance associates with various diseases^17^.

The role of genetic in SCZ pathophysiology is still vague. The recently identified molecular mechanisms regulating gene transcription could help elucidate how the alterations in gene expression could affect SCZ development and progression. Thereby more efficient therapeutic and diagnostic approaches could be found. Comparative analysis of gene expression profiles between patients and controls provides insights into exploring pathophysiological mechanisms and helps identify potential biomarkers^18^. Uncovering the molecular mechanisms behind psychiatric disorders’ pathogenesis is challenging due to the difficulty of accessing central nervous system tissues and cells from live patients. While post-mortem brain samples are invaluable for molecular studies, these samples do not appear to provide reliable molecular information related to the onset or course of cognitive deficits within the same living subjects. Thereby, reliable biological specimens and also samples that can be obtained longitudinally are required^19,20^. Although blood sample is frequently utilized and easy to obtain, it has been revealed that blood cells and brain cells differ in gene expression profile in SCZ studies. It is noteworthy that olfactory epithelium (OE) contains olfactory receptor neurons, which show similar expression patterns to developing brain cells^19–21^. One interesting element of employing OE tissues in the field of psychiatric illness is their link with the wider olfactory neurocircuitry: olfactory mucosa → olfactory bulb → olfactory cortex^22^. Alterations in the OE as a result of the disease may not arise in isolation and may represent brain abnormalities in the wider olfactory neurocircuitry. Many structural and functional changes linked with neuropsychiatric illnesses have been observed in most of these brain areas. Therefore, OE is a better choice for molecular studies of SCZ patients^19–22^.

In this study, we performed a bioinformatics analysis to identify lncRNA-associated ceRNA axes in the OE of live SCZ patients to elucidate molecular regulatory mechanisms related to the disease.

## Methods

**Figure 1:**
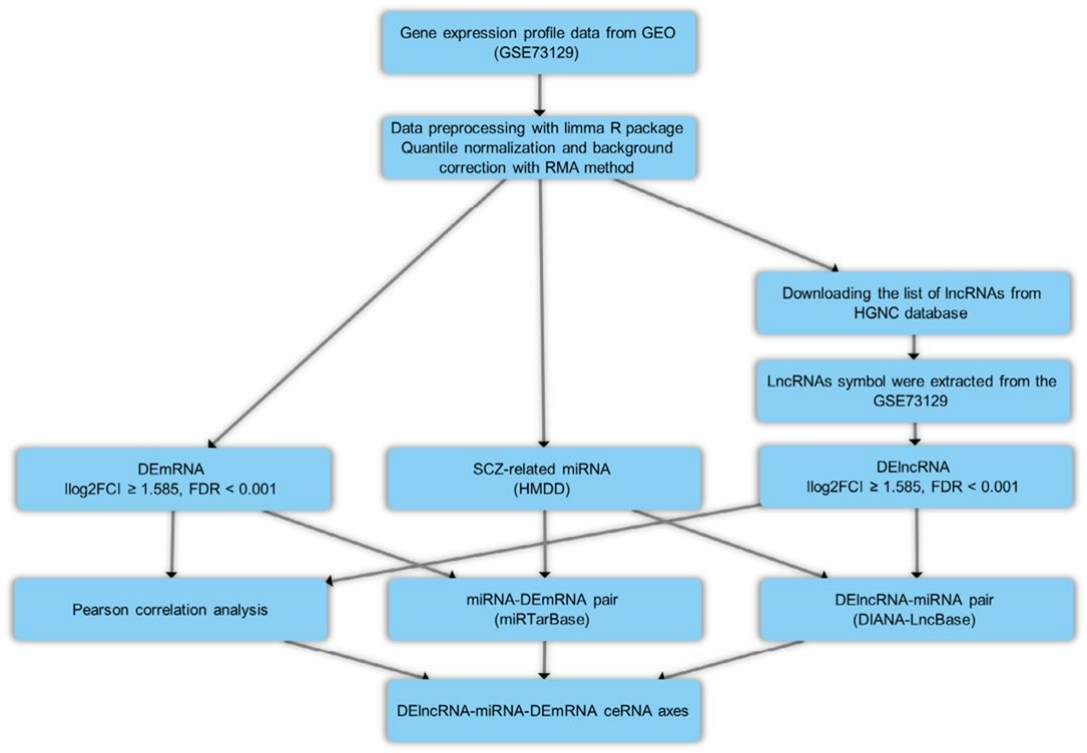
Flow chart of bioinformatics analysis.

In the present study, we utilized a bioinformatics approach for data mining of the microarray dataset (GSE73129) with the olfactory epithelium (OE) biopsy samples from SCZ patients and matched controls. We intended to identify differentially expressed mRNAs (DEmRNAs) as well as lncRNAs (DElncRNAs) and construct lncRNA-associated ceRNA regulatory axes. Figure 1 summarizes the stages performed in the bioinformatics strategy.

### Gene expression profile data collection

The gene expression profile mentioned above was collected from the NCBI Gene Expression Omnibus database (GEO, https://www.ncbi.nlm.nih.gov/geo/). The microarray dataset was based on the GPL570 platform (Affymetrix Human Genome U133 Plus 2.0 Array). The GPL570 contains both mRNA and lncRNA information. The GSE73129 dataset contains 19 OE tissues collected from SCZ patients and 19 OE tissues from healthy individuals^20^.

### Data preprocessing and DEmRNAs and DElncRNAs identification

For background correction and quantile normalization of all primary data records, Robust Multichip Average (RMA) was applied^23^. An interquartile range filter (IQR across the samples on the log base two scale greater than median IQR) was carried out, which was accompanied by an intensity filter (a minimum of > 100 expression signals in a minimum of 25% of the arrays) intending to eliminate insignificant probe sets that are not expressed^24^. For quality control, the AgiMicroRna bioconductor package was applied. We performed principal component analysis (PCA) to conduct a dimensional reduction analysis^25^, aiming to find similarities between each sample group using the ggplot2 package of R software version 4.0.3 (https://www.r-project.org/)^26^. Differential expression gene analysis (DEGA) was done between SCZ and normal samples using the linear models for microarray data (limma) R package^27^ in bioconductor (https://www.bioconductor.org/)^28^. We utilized the previously used approach to identify lncRNA probes^29^. Then the complete list of lncRNA genes with HUGO Gene Nomenclature Committee (HGNC) approved symbols were retrieved from the HGNC database (https://www.genenames.org/)^30^. Afterward, we compared the lncRNA gene list with our dataset gene symbols and chose the overlapped genes. We used the student t-test and the aberrantly expressed RNAs cut-off were set as follows: (1) a false discovery rate (adjusted *P* value) < 0.001, and (2) |log2 fold change (log2FC) |> 1.585. There is currently no gold standard for selecting fold change and adjusted *P* value cut-off. We filtered DEGs using stringent criteria to minimize false positives and analyze genes that had a drastic increase or decrease. It has been proven that biological changes caused by ceRNA regulation are only observable when the miRNA/ceRNA levels increase or decrease drastically in certain physiological states^31^. The Pheatmap and Enhanced Volcano R packages were used to draw the DEGs’ heat map and volcano plot.

### Prediction of RNA interaction pairs

The experimentally validated interactions between miRNAs and DElncRNAs were identified using DIANA-LncBase v3^32^. Homo Sapiens “Species” and high “miRNA Confidence Levels” were considered as criteria for the DIANA-LncBase query. SCZ-related miRNAs were collected from the Human microRNA Disease Database (HMDD) v3.2 database^33^. Furthermore, we retrieved the interactions between miRNAs that were collected using the HMDD and target mRNAs from miRTarBase^34^, supported by strong experimental evidence. After comparing the retrieved mRNAs and the previously obtained mRNAs, the duplicated mRNAs were utilized to construct the DElncRNA-miRNA-DEmRNA regulatory axes.

### Correlation analysis between DElncRNAs and DEmRNAs, and lncRNA-associated ceRNA axes construction

The Pearson correlation analysis was performed in order to assess positive correlations between DElncRNAs and DEmRNAs in the ceRNA regulatory axes. DELncRNAs, targeted DEmRNAs, and the interacted miRNAs were removed from the ceRNA network in the opposite expression pattern between the targeted DEmRNAs and DElncRNAs. The Hmisc and corrplot packages were applied to calculate the correlations and visualization. Pearson correlation coefficient > 0.5 and P < 0.001 were used as inclusion criteria. Cytoscape software (version 3.8.0) (https://cytoscape.org/)^35^ was applied to construct the ceRNA regulatory axes.

## Results

### DEmRNAs and DElncRNAs identification

Background adjustment, normalization, gene filtering, and batch adjustment were done before performing DEGA. To control the quality, the AgiMicroRna bioconductor package was used. Following normalizing, box plots for the gene expression data were illustrated to analyze data distribution (Supplementary File S1). Separate arrays in the box plots showed identical medians of expression level, indicating correct adjustment. Furthermore, PCA plot was used to show the spatial distribution of samples (Supplementary File S1). The details of the examined data structure are displayed in PCA. Also, it helps assess similarities between samples. Two control samples were removed due to being spatially far from other control samples.

**Figure 2:**
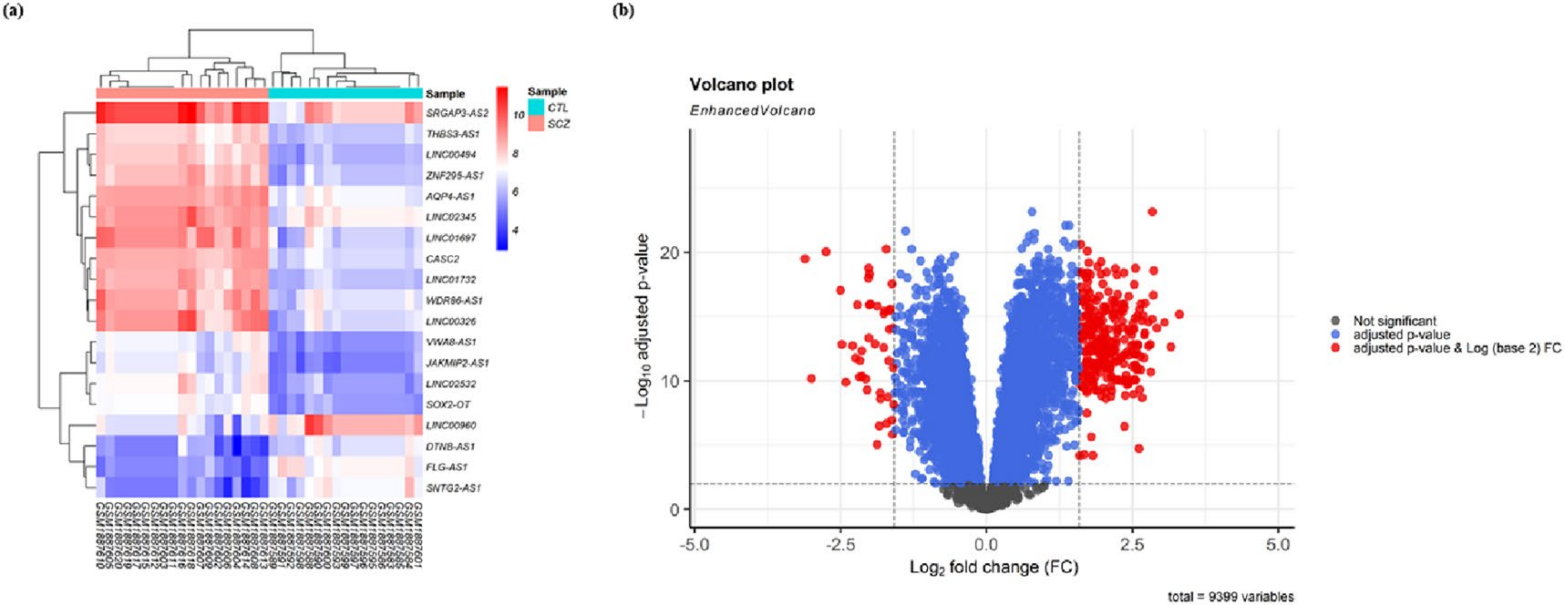
Differentially expressed lncRNAs (DElncRNAs) and mRNAs (DEmRNAs) between schizophrenia (SCZ) samples and control (CTL) samples. (**a**) Hierarchical clustering heatmap of DElncRNAs. High expressed lncRNAs are shown in red, while those expressed at low levels are blue. (**b**) Volcano plot for the DEmRNAs. The DElncRNAs and DEmRNAs were screened according to a |(log2FC)|> 1.585 and an adjusted *P* value < 0.001. This figure was made using Pheatmap and Enhanced Volcano packages of R version 4.0.3 (https://www.r-project.org/).

Based on the stringent criteria (adjusted *P* value < 0.001, and (2) |log2 fold change (log2FC) |> 1.585), a total of 19 DElncRNAs and 303 DEmRNAs were identified in GSE73129 between SCZ and healthy control OE samples. Hierarchical clustering heatmap of DElncRNAs and volcano plot of DEmRNAs are shown in Fig. 2. The details of DEGs are summarized in Supplementary File S2.

### Prediction of RNA interaction pairs

We applied the DIANA-LncBase v3 online tool and predicted DElncRNA-miRNA interaction pairs based on the DElncRNAs and consequently revealed that 13 of the 19 DElncRNAs might target candidate miRNAs. Subsequently, miRTarBase was used to find the interactions between miRNAs that were collected using the HMDD and candidate mRNAs. Following a comparison between the candidate mRNAs with 304 DEmRNAs, we identified seven overlapping genes.

### Correlation analysis between DElncRNAs and DEmRNAs, and lncRNA-associated ceRNA axes construction

**Figure 3:**
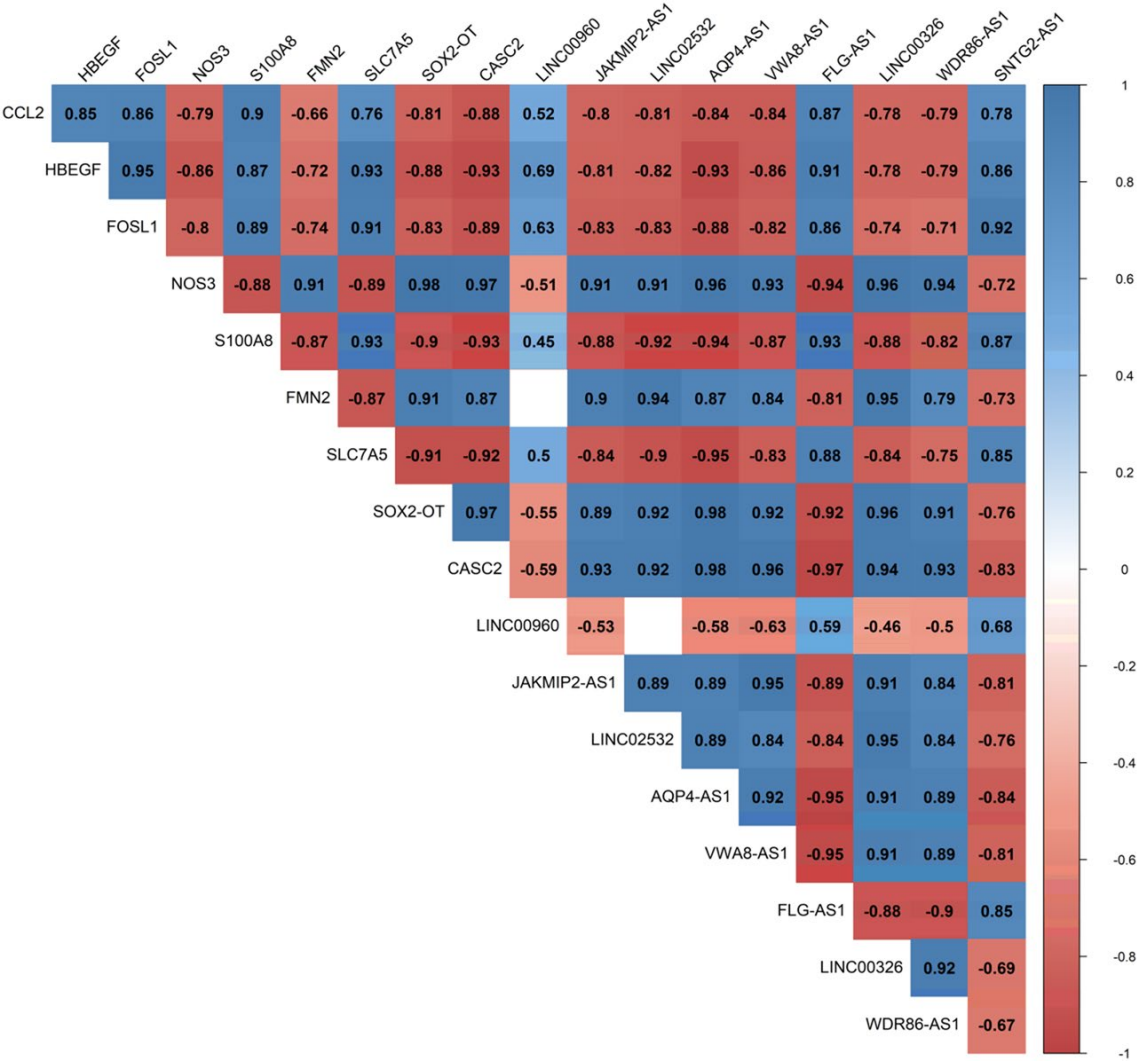
Positive correlations are shown in blue, while negative correlations are shown in red. The intensity of the colors is related to correlation coefficients, and the ones with a *P* value greater than 0.001 are deemed insignificant. Note that values of correlation coefficients are left blank in this situation. This figure was made using Hmisc and corrplot packages of R version 4.0.3 (https://www.r-project.org/).

**Figure 4:**
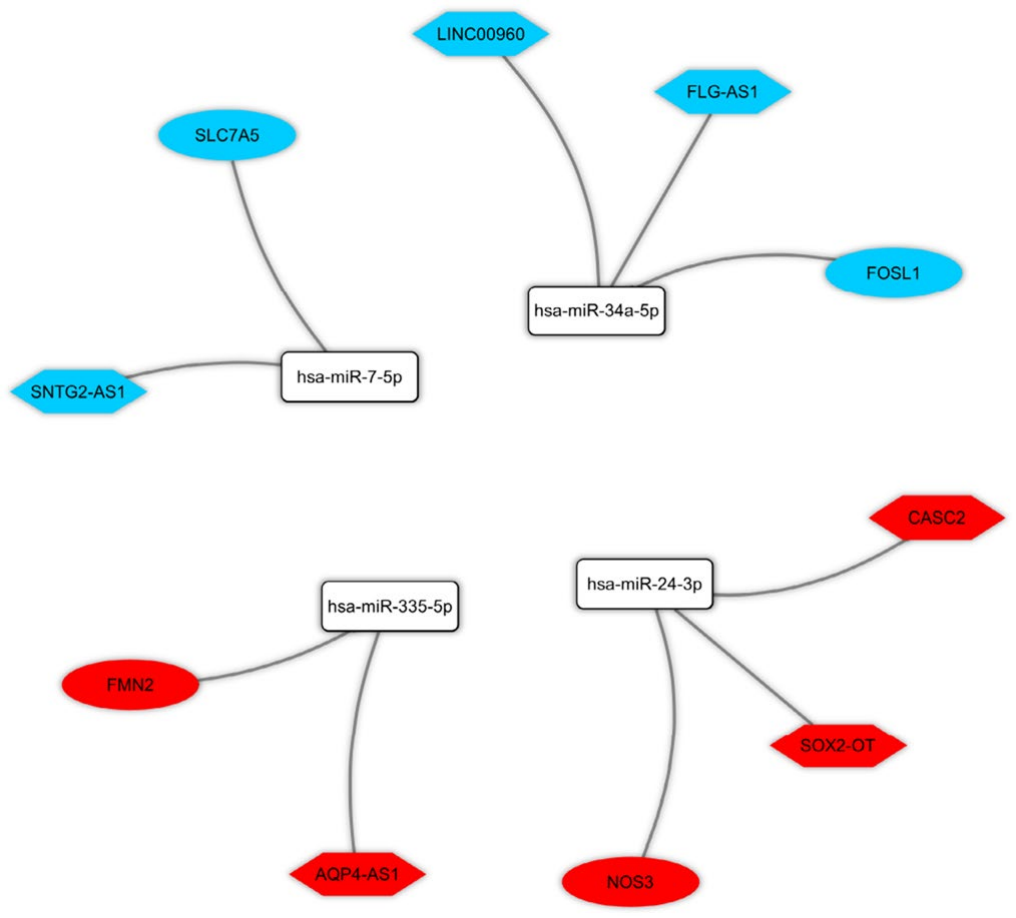
The long non-coding RNA-associated competing endogenous RNA (ceRNA) axes in OE in schizophrenia. The red and blue nodes represent the upregulation and downregulation, respectively. Gray edges represent interactions between RNAs. LncRNAs, miRNAs, and mRNAs are represented by hexagon, round rectangle, and ellipse, respectively.

The Pearson correlation analysis was performed between DElncRNAs and DEmRNAs to verify the ceRNA axes hypothesis, which mRNA expression is positively regulated by lncRNA through interaction with miRNA (Fig. 3). Based on the co-expression relationships and DElncRNA-miRNA-DEmRNA interactions, we constructed ceRNA regulatory axes to elucidate the mechanism underlying the pathogenesis of SCZ (Fig. 4). In total, six DElncRNAs (*SNTG2-AS1*: SNTG2 antisense RNA 1, *FLG-AS1*: FLG antisense RNA 1, *LINC00960*: long intergenic non-protein coding RNA 960, *AQP4-AS1*: AQP4 antisense RNA 1, *SOX2-OT*: SOX2 overlapping transcript, and *CASC2*: cancer susceptibility 2), four miRNAs (*hsa-miR-34a-5p*, *hsa-miR-7-5p*, *hsa-miR-335-5p*, and *hsa-miR-24-3p*), and four DEmRNAs (*FOSL1*: FOS like 1, *SLC7A5*: solute carrier family 7 member 5, *FMN2*: formin 2, *NOS3*: nitric oxide synthase 3) were included.

## Discussion

Emerging evidence shows deficits in olfactory function in various neuropsychiatric disorders such as SCZ, Alzheimer’s, and Parkinson’s disease. This might be related to cellular or molecular alterations in the OE^22^, which harbors neuronal lineage cells at various stages of maturation^36,37^. The OE model provides a lot of advantages. First, ex-vivo OE tissues were exposed previously to the in-vivo neurohormonal milieu, having in-vivo neurobiological hallmarks. Moreover, they can act as a reference for both in vitro and in vivo OE results, bridging the gap between the two techniques. Second, while restricted by the amount of tissue accessible, OE biopsies may safely be taken numerous times from the same patients and can be integrated with longitudinal clinical research designs. OE biopsies are collected in certain stages of the disease in this procedure, while patients' clinical circumstances are meticulously documented^22^. It is a unique chance in neuropsychiatric research to interpret neurobiological variables regarding clinical alterations. Examples of such applications comprise, but are not confined to, collecting and evaluating OE biopsies from people at risk for the onset of schizophrenia and their intact relatives prior to and after the development of disease^22^.

A number of studies have demonstrated the activity of ceRNA regulatory loops and the related networks in several pathological conditions and developmental processes, e.g., tumorigenesis, neurodegenerative diseases^38^, and mental disorders^39,40^. There may be various ceRNAs, including mRNAs, pseudogenes, circRNAs, and lncRNAs, in a network^31^. LncRNAs, as a major group of RNAs within the ceRNA machinery, have a key role in the regulation of pathological and physiological cellular mechanisms. The expression of lncRNAs found to be affected under various mental conditions^41^. Interestingly, lncRNAs expression depends on developmental level, cell and tissue type. Subcellular distributions and tissue specificity indicate intensive regulation of lncRNAs expression^42^. According to the above-mentioned theoretical concepts, the lncRNA-related ceRNA regulatory network can substantially affect SCZ pathogenesis. Studies on SCZ- associated ceRNA regulatory loops remain yet to be extended, and it is necessary to further examine the corresponding mechanisms and patterns of expression in SCZ. In the present study, the OE sample expression profiles were downloaded from a public database to evaluate DEmRNAs and DElncRNAs in SCZ and normal tissues in order to construct DElncRNA-miRNA-DEmRNA regulatory loops. We identified six possible DElncRNA-miRNA-DEmRNA loops in the pathogenesis of SCZ: *SNTG2-AS1/hsa-miR-7-5p/SLC7A5, FLG-AS1/hsa-miR-34a-5p/FOSL1, LINC00960/hsa-miR-34a-5p/FOSL1, AQP4-AS1/hsa-miR-335-5p/FMN2, SOX2-OT/hsa-miR-24-3p/NOS3, and CASC2/hsa-miR-24-3p/NOS3*.

A number of studies have suggested that dysregulation of lncRNAs might participate in the pathogenesis of SCZ^43^. The current study identified several DElncRNAs, among which only the association between *SOX2-OT* and SCZ was identified in previous studies. *SOX2-OT* is an evolutionarily conserved lncRNA. *SOX2* gene, an essential embryonic stem cell pluripotency regulator, is embedded in intronic region of *SOX2-OT*. The *SOX2-OT*, as an important ceRNA, has been identified to influence the progression of multiple cancers. According to genome-wide association studies (GWAS), there are associations between mental disorders (e.g., general cognitive disorders, SCZ, eating disorders, insomnia, anorexia nervosa, and night sleep phenotypes) and *SOX-OT*-mapped single nucleotide polymorphisms (SNPs). Mental conditions account for over half of *SOX2-OT*-related disorders^44^. *FLG-AS1* and *AQP4-AS1* are two other DElncRNAs that were identified by our analysis. Dysregulation of *FLG-AS1* was reported in some cancers, but the specific function and detailed mechanisms of *FLG‐AS1* are still unknown^45,46^. A previous integrative analysis showed that *FLG‐AS1* acts as a ceRNA in adipose tissue from obese individuals with type 2 diabetes^47^. Earlier studies have found that structural variant within *FLG‐AS1* may play an important role in attention-deficit hyperactivity disorder (ADHD) development^48^. The *AQP4-AS1* gene transcribes an antisense lncRNA with an unknown function^49^. *AQP4-AS1* was identified as a ceRNA in gastric cancer via bioinformatics analysis^50^. Moreover, a previous study reported that *AQP4-AS1* is associated with depression as a mental disorder^51^. To the best of our knowledge, the association between *SNTG2-AS1*, *CASC2*, and *LINC00960* lncRNAs and mental disorders has not been studied thus far. *SNTG2-AS1* is an antisense lncRNA, and its function is still unknown^52^. Since the host transcript could be regulated by the same number of antisense transcripts^53^, the contributions of this lncRNA may be realized by the nearby syntrophin gamma 2 (*SNTG2*) gene. *SNTG2* encoded protein is a member of the syntrophin family. Syntrophins are crucial scaffolding proteins, due to binding to the dystrobrevin and dystrophin^54^. The interrupted 2p25.3 duplication encompassing *SNTG2* was identified in two SCZ patients^55^. The biological functions of *LINC00960*, as a newly discovered lncRNA, in human diseases remain to be elucidated. It was indicated that *LINC00960* acts as a ceRNA in diabetic nephropathy and pancreatic ductal adenocarcinoma^56,57^. As a lncRNA, *CASC2* suppresses tumors in various tissues and influences multiple signaling pathways and genes. It plays a role as a ceRNA for some miRNAs and influences the activity of their targets^58^.

MiRNAs bind to the untranslated region of the target gene, consequently controlling the target gene expression. It has been identified that miRNAs can affect signal transduction and biological pathways within the cell and induce SCZ progression^59^. The current study revealed that the key DElncRNAs could sponge four important miRNAs (*hsa-miR-34a-5p*, *hsa-miR-7-5p*, *hsa-miR-335-5p*, and *hsa-miR-24-3p*) that are associated with SCZ, resulting in the regulation of key DEmRNAs. In line with our findings, increased expressions of *hsa-miR-34a-5p* and *hsa-miR-7-5p* in the periphery of SCZ patients^60–62^ and decreased expressions of *hsa-miR-24-3p* in the prefrontal cortex of affected individuals^63^ have been reported previously. Besides, previous studies of the miRNA-derived network analysis reported the fine-tuning of the genes involved in the SCZ biological pathway by *hsa-miR-335-5p*. Hence, they supported the ceRNA emerging theory^64^. The findings of the present work are supported by most of such works; however, molecular methods (e.g., PCR, co-immunoprecipitation assays, and luciferase reporter systems) are yet to be employed to validate the predicted ceRNA loops.

Four key DEmRNAs (*FOSL1*, *SLC7A5*, *FMN2*, and *NOS3*) were reported in this study. *FOS*, *FOSB*, *FOSL1*, and *FOSL2* are the FOS family members. This family encodes the leucine zipper proteins, which have the capability of dimerization with JUN, JUND, and JUNB (the JUN family members) in order to form the AP-1 transcription factor. Hence, the FOS family regulates the proliferation, transformation, differentiation, and apoptotic death of cells^65^. Based on upstream transcription factor analysis and high-throughput gene studies, *FOSL1* implicates in SCZ through various mechanisms, such as influencing accessible chromatin regions and connecting to neuroinflammation signaling cascades^66–68^. *SLC7A5*, also known as *LAT1*, encodes a large amino acid transporter located in the blood–brain barrier. It is essential in the maintenance of brain branched-chain amino acids' normal levels^69^. It was revealed that SCZ patients have an aberrant amino acid transport activities, such as the aberrant tyrosine transport through the plasma membrane^70^ and excitatory amino acid transport^71^. Moreover, genetic and functional studies of the *SLC7A5* gene have suggested an association between *SLC7A5* SNP rs9936204 genotype with SCZ vulnerability in humans, and also, the SLC7A5 isoform was identified to be a significant transporter of tyrosine in SCZ patients^72^. The formin family is a significant effector class for not only microtubule regulation but also actin regulation. This family includes proteins containing the formin homology 1 (FH1) and formin homology 2 (FH2) domains. *FMN2* is a member of the formin family and is highly expressed in several regions of the developing and adult brain as well as the spinal cord. The major characteristic of FMN2 is its contribution to actin dynamics regulation. It is an interesting regulator of actin in Wnt canonical pathway homeostatic regulation within neural progenitors. FMN2 not only regulates actin dynamics and bundling but also associates with axon growth cone maintenance and pathfinding. Previous findings have associated high *FMN2* expression maintenance within differentiated neurons with the maintenance and plasticity of the synapse^73^. It has been reported that formin genes are involved in a number of neural disorders (e.g., amyotrophic lateral sclerosis^74^ and SCZ^75,76^). The *FMN2* SNPs rs6050455 and rs6656902 were known to be associated with SCZ^77,78^. *NOS3* belongs to the nitric oxide synthase (NOS) enzyme family and participates in the nitric oxide (NO) generation. Research has shown that NO contributes to SCZ pathogenesis. There is solid evidence of the effects of the NO metabolism on SCZ processes, including nerve cell migration, synapse formation and maintenance, N-methyl-D-aspartic acid receptor-mediated neurotransmission, cognitive abilities, membrane pathology, and hippocampal neurogenesis^79^. Moreover, there is evidence that the SNPs of *NOS3* are associated with SCZ^80,81^.

It is noteworthy that several technical factors, such as different methodologies, patient characteristics, preparation of samples, analysis of data, and platforms, could affect the gene expression profiles. Of course, confirmative experimental works and comparisons to reanalysis modified microarray gene expression are required to validate our findings.

## Conclusion

In conclusion, we identified six possible DElncRNA-miRNA-DEmRNA loops in OE tissues of living SCZ patients, including, *SNTG2-AS1/hsa-miR-7-5p/SLC7A5, FLG-AS1/hsa-miR-34a-5p/FOSL1, LINC00960/hsa-miR-34a-5p/FOSL1, AQP4-AS1/hsa-miR-335-5p/FMN2, SOX2-OT/hsa-miR-24-3p/NOS3, and CASC2/hsa-miR-24-3p/NOS3*. Although the potential functions of these ceRNAs are required to be further investigated, the current study presents a new perspective into the molecular mechanisms behind SCZ pathogenesis that might help elucidate the different aspects of neurodevelopment that may have been hampered early in SCZ patients.

## Supplementary Information

Supplementary Information 1.

Supplementary Information 2.

## References

[1] Kahn RS (2015). Schizophrenia. Nat. Rev. Dis. Primers.

[2] Falkai P (2015). Kraepelin revisited: Schizophrenia from degeneration to failed regeneration. Mol. Psychiatry.

[3] Davarinejad O (2021). Identification of risk factors to predict the occurrences of relapses in individuals with schizophrenia spectrum disorder in Iran. Int. J. Environ. Res. Public Health.

[4] Canitano R, Pallagrosi M (2017). Autism spectrum disorders and schizophrenia spectrum disorders: Excitation/inhibition imbalance and developmental trajectories. Front. Psychiatry.

[5] Alizadeh M (2021). Non-linear associations between retinal nerve fibre layer (RNFL) and positive and negative symptoms among men with acute and chronic schizophrenia spectrum disorder. J. Psychiatric Res..

[6] Farnia V (2020). Comparisons of voxel-based morphometric brain volumes of individuals with methamphetamine-induced psychotic disorder and schizophrenia spectrum disorder and healthy controls. Neuropsychobiology.

[7] Narayan S (2008). Molecular profiles of schizophrenia in the CNS at different stages of illness. Brain Res..

[8] Roy M (2018). Proteomic analysis of postsynaptic proteins in regions of the human neocortex. Nat. Neurosci..

[9] Ramaker RC (2017). Post-mortem molecular profiling of three psychiatric disorders. Genome Med..

[10] Ghafouri-Fard S (2021). A review on the expression pattern of non-coding RNAs in patients with schizophrenia: With a special focus on peripheral blood as a source of expression analysis. Front. Psychiatry.

[11] Gibbons A, Udawela M, Dean B (2018). Non-coding RNA as novel players in the pathophysiology of schizophrenia. Noncoding RNA.

[12] Beveridge NJ, Gardiner E, Carroll AP, Tooney PA, Cairns MJ (2010). Schizophrenia is associated with an increase in cortical microRNA biogenesis. Mol. Psychiatry.

[13] Santarelli DM, Beveridge NJ, Tooney PA, Cairns MJ (2011). Upregulation of dicer and microRNA expression in the dorsolateral prefrontal cortex Brodmann area 46 in schizophrenia. Biol. Psychiatry.

[14] Meng Q (2018). The DGCR5 long noncoding RNA may regulate expression of several schizophrenia-related genes. Sci. Transl. Med..

[15] Safari MR, Komaki A, Arsang-Jang S, Taheri M, Ghafouri-Fard S (2019). Expression pattern of long non-coding RNAs in schizophrenic patients. Cell Mol. Neurobiol..

[16] Salmena L, Poliseno L, Tay Y, Kats L, Pandolfi PP (2011). A ceRNA hypothesis: The rosetta stone of a hidden RNA language?. Cell.

[17] Sen R, Ghosal S, Das S, Balti S, Chakrabarti J (2014). Competing endogenous RNA: The key to posttranscriptional regulation. TheScientificWorldJOURNAL.

[18] Iwamoto K, Kato T (2006). Gene expression profiling in schizophrenia and related mental disorders. Neuroscientist.

[19] Horiuchi Y (2013). Olfactory cells via nasal biopsy reflect the developing brain in gene expression profiles: Utility and limitation of the surrogate tissues in research for brain disorders. Neurosci. Res..

[20] Horiuchi Y (2016). Molecular signatures associated with cognitive deficits in schizophrenia: A study of biopsied olfactory neural epithelium. Transl. Psychiatry.

[21] Cascella NG, Takaki M, Lin S, Sawa A (2007). Neurodevelopmental involvement in schizophrenia: The olfactory epithelium as an alternative model for research. J. Neurochem..

[22] Borgmann-Winter K (2015). Translational potential of olfactory mucosa for the study of neuropsychiatric illness. Transl. Psychiatry.

[23] Irizarry RA (2003). Exploration, normalization, and summaries of high density oligonucleotide array probe level data. Biostatistics (Oxford, England).

[24] von Heydebreck A, Huber W, Gentleman R (2005). Encyclopedia of Genetics, Genomics, Proteomics and Bioinformatics.

[25] Yeung KY, Ruzzo WL (2001). Principal component analysis for clustering gene expression data. Bioinformatics (Oxford, England).

[26] Wickham H (2016). ggplot2-Elegant Graphics for Data Analysis.

[27] Ritchie ME (2015). Limma powers differential expression analyses for RNA-sequencing and microarray studies. Nucl. Acids Res..

[28] Huber W (2015). Orchestrating high-throughput genomic analysis with bioconductor. Nat. Methods.

[29] Dashti S, Taheri M, Ghafouri-Fard S (2020). An in-silico method leads to recognition of hub genes and crucial pathways in survival of patients with breast cancer. Sci. Rep..

[30] Tweedie S (2021). Genenames.org: The HGNC and VGNC resources in 2021. Nucl. Acids Res..

[31] Cai Y, Wan J (2018). Competing endogenous RNA regulations in neurodegenerative disorders: Current challenges and emerging insights. Front. Mol. Neurosci..

[32] Karagkouni D (2020). DIANA-LncBase v3: Indexing experimentally supported miRNA targets on non-coding transcripts. Nucl. Acids Res..

[33] Huang Z (2019). HMDD v3.0: A database for experimentally supported human microRNA-disease associations. Nucl. Acids Res..

[34] Huang HY (2020). miRTarBase 2020: Updates to the experimentally validated microRNA-target interaction database. Nucl. Acids Res..

[35] Shannon P (2003). Cytoscape: A software environment for integrated models of biomolecular interaction networks. Genome Res..

[36] Leung CT, Coulombe PA, Reed RR (2007). Contribution of olfactory neural stem cells to tissue maintenance and regeneration. Nat. Neurosci..

[37] Schwob JE (2002). Neural regeneration and the peripheral olfactory system. Anat. Rec..

[38] Ala U (2020). Competing endogenous RNAs, non-coding RNAs and diseases: An intertwined story. Cells.

[39] Lang Y, Zhang J, Yuan Z (2019). Construction and dissection of the ceRNA-ceRNA network reveals critical modules in depression. Mol. Med. Rep..

[40] Li Z (2020). Circular RNA in schizophrenia and depression. Front. Psychiatry..

[41] Zuo L (2016). Long noncoding RNAs in psychiatric disorders. Psychiatr. Genet..

[42] Gloss BS, Dinger ME (1859). The specificity of long noncoding RNA expression. Biochimica et Biophysica Acta (BBA) - Gene Regulatory Mechanisms.

[43] Wang Z, Tong Q, Liao H, Rao S, Huang X (2018). Long non-coding RNAs in schizophrenia. Neurol. Psychiatry Brain Res..

[44] Li P-Y, Wang P, Gao S-G, Dong D-Y (2020). Long Noncoding RNA SOX2-OT: Regulations, functions, and roles on mental illnesses, cancers, and diabetic complications. Biomed. Res. Int..

[45] Maimaiti A (2021). Identification and validation of a novel eight mutant-derived long non-coding RNAs signature as a prognostic biomarker for genome instability in low-grade glioma. Aging (Albany NY).

[46] Zhang C (2020). A three-lncRNA signature of pretreatment biopsies predicts pathological response and outcome in esophageal squamous cell carcinoma with neoadjuvant chemoradiotherapy. Clin. Transl. Med..

[47] Deng G (2020). Circular RNA circRHOBTB3 acts as a sponge for miR-654-3p inhibiting gastric cancer growth. J. Exp. Clin. Cancer Res..

[48] Liu Y (2020). Non-coding structural variation differentially impacts attention-deficit hyperactivity disorder (ADHD) gene networks in African American vs Caucasian children. Sci. Rep..

[49] Halladay JR (2018). Applicability of precision medicine approaches to managing hypertension in rural populations. J. Pers. Med..

[50] Xing C (2018). Identification of potential biomarkers involved in gastric cancer through integrated analysis of non-coding RNA associated competing endogenous RNAs network. Clin. Lab..

[51] Westermair AL (2018). Association of genetic variation at AQP4 locus with vascular depression. Biomolecules.

[52] Stelzer G (2016). The genecards suite: From gene data mining to disease genome sequence analyses. Curr. Protocols Bioinform..

[53] Wight M, Werner A (2013). The functions of natural antisense transcripts. Essays Biochem..

[54] Adams ME (2004). Structural abnormalities at neuromuscular synapses lacking multiple syntrophin isoforms. J. Neurosci..

[55] Van Den Bossche MJ (2013). Identification of rare copy number variants in high burden schizophrenia families. Am. J. Med. Genet. Neuropsychiatr. Genet..

[56] Yu Y, Jia YY, Wang M, Mu L, Li HJ (2021). PTGER3 and MMP-2 play potential roles in diabetic nephropathy via competing endogenous RNA mechanisms. BMC Nephrol..

[57] Huang Y (2021). Long intergenic non-protein coding RNA 960 regulates cancer cell viability, migration and invasion through modulating miR-146a-5p/interleukin 1 receptor associated kinase 1 axis in pancreatic ductal adenocarcinoma. Bioengineered.

[58] Ghafouri-Fard S, Dashti S, Taheri M (2020). The role of long non-coding RNA CASC2 in the carcinogenesis process. Biomed. Pharmacother..

[59] Caputo V, Ciolfi A, Macri S, Pizzuti A (2015). The emerging role of MicroRNA in schizophrenia. CNS Neurol. Disord. Drug. Targets.

[60] Lai C-Y (2011). MicroRNA expression aberration as potential peripheral blood biomarkers for schizophrenia. PLoS ONE.

[61] Sun XY (2015). Aberrant microRNA expression in peripheral plasma and mononuclear cells as specific blood-based biomarkers in schizophrenia patients. J. Clin. Neurosci..

[62] Choi S-Y (2015). Post-transcriptional regulation of SHANK3 expression by microRNAs related to multiple neuropsychiatric disorders. Mol. Brain.

[63] Perkins DO (2007). microRNA expression in the prefrontal cortex of individuals with schizophrenia and schizoaffective disorder. Genome Biol..

[64] Gumerov V, Hegyi H (2015). MicroRNA-derived network analysis of differentially methylated genes in schizophrenia, implicating GABA receptor B1 [GABBR1] and protein kinase B [AKT1]. Biol. Direct..

[65] Jin Y (2011). Molecular characterization of the microRNA-138-Fos-like antigen 1 (FOSL1) regulatory module in squamous cell carcinoma. J. Biol. Chem..

[66] Curtis D (2016). Pathway analysis of whole exome sequence data provides further support for the involvement of histone modification in the aetiology of schizophrenia. Psychiatr. Genet..

[67] Bryois J (2018). Evaluation of chromatin accessibility in prefrontal cortex of individuals with schizophrenia. Nat. Commun..

[68] Izumi R (2021). Detailed postmortem profiling of inflammatory mediators expression revealed post-inflammatory alternation in the superior temporal gyrus of schizophrenia. Front. Psychiatry..

[69] Guan J, Cai JJ, Ji G, Sham PC (2019). Commonality in dysregulated expression of gene sets in cortical brains of individuals with autism, schizophrenia, and bipolar disorder. Transl. Psychiatry.

[70] Flyckt L (2001). Aberrant tyrosine transport across the cell membrane in patients with schizophrenia. Arch. Gen. Psychiatry.

[71] Smith RE, Haroutunian V, Davis KL, Meador-Woodruff JH (2001). Expression of excitatory amino acid transporter transcripts in the thalamus of subjects with schizophrenia. Am. J. Psychiatry.

[72] Comasco E (2016). Genetic and functional study of L-type amino acid transporter 1 in schizophrenia. Neuropsychobiology.

[73] Kawabata Galbraith K, Kengaku M (2019). Multiple roles of the actin and microtubule-regulating formins in the developing brain. Neurosci. Res..

[74] Schymick JC (2007). Genome-wide genotyping in amyotrophic lateral sclerosis and neurologically normal controls: First stage analysis and public release of data. Lancet Neurol..

[75] Proitsi P (2008). Positional pathway screen of wnt signaling genes in schizophrenia: Association with DKK4. Biol. Psychiatry.

[76] Kuzman MR, Medved V, Terzic J, Krainc D (2009). Genome-wide expression analysis of peripheral blood identifies candidate biomarkers for schizophrenia. J. Psychiatric Res..

[77] van Scheltinga AFT (2013). Genetic schizophrenia risk variants jointly modulate total brain and white matter volume. Biol. Psychiatry.

[78] Lee K-Y (2020). Genome-wide search for SNP interactions in GWAS data: Algorithm, feasibility, replication using schizophrenia datasets. Front. Genet..

[79] Bernstein HG, Keilhoff G, Steiner J, Dobrowolny H, Bogerts B (2011). Nitric oxide and schizophrenia: Present knowledge and emerging concepts of therapy. CNS Neurol. Disord. Drug Targets.

[80] Liou YJ (2006). Haplotype analysis of endothelial nitric oxide synthase (NOS3) genetic variants and tardive dyskinesia in patients with schizophrenia. Pharmacogenet. Genomics.

[81] Thelma BK, Tiwari AK, Deshpande SN, Lerer B, Nimgaonkar VL (2007). Genetic susceptibility to tardive dyskinesia in chronic schizophrenia subjects: Role of oxidative stress pathway genes. Schizophr. Res..

