# Supplementary material for: Long non-coding RNA-associated competing endogenous RNA axes in the olfactory epithelium in schizophrenia: a bioinformatics analysis
Source: Sci Rep. 2021 Dec 30;11:24497. doi: 10.1038/s41598-021-04326-0 (PMC8718521; doi:10.1038/s41598-021-04326-0)
Supplement: Supplementary file 1 — Supplementary Information 1. [file 41598_2021_4326_MOESM1_ESM.docx]

**
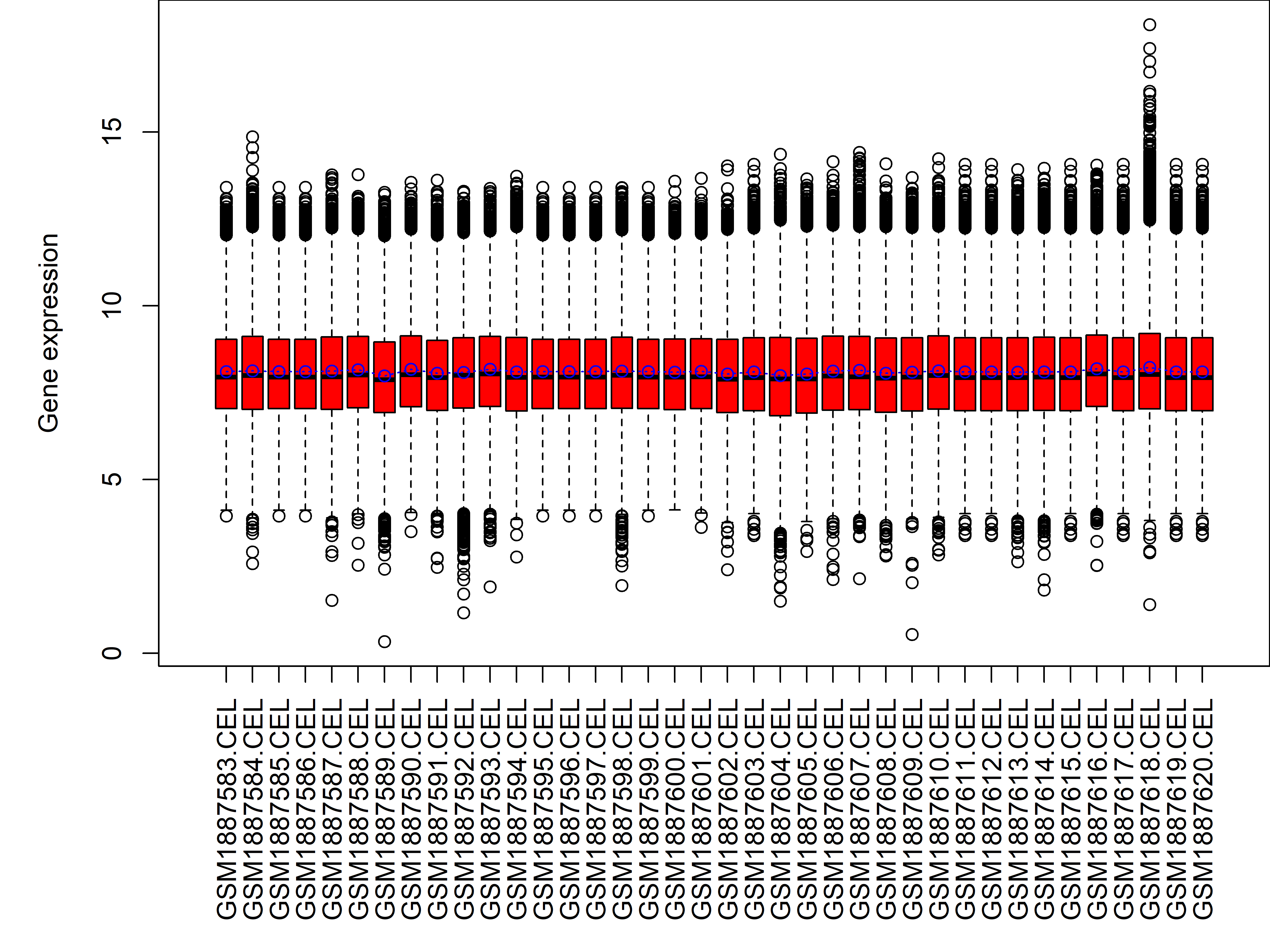
**

**Figure S1.** Boxplot for GSE73129 dataset. The horizontal axis represents the names of samples, and the vertical axis represents the gene expression.

**
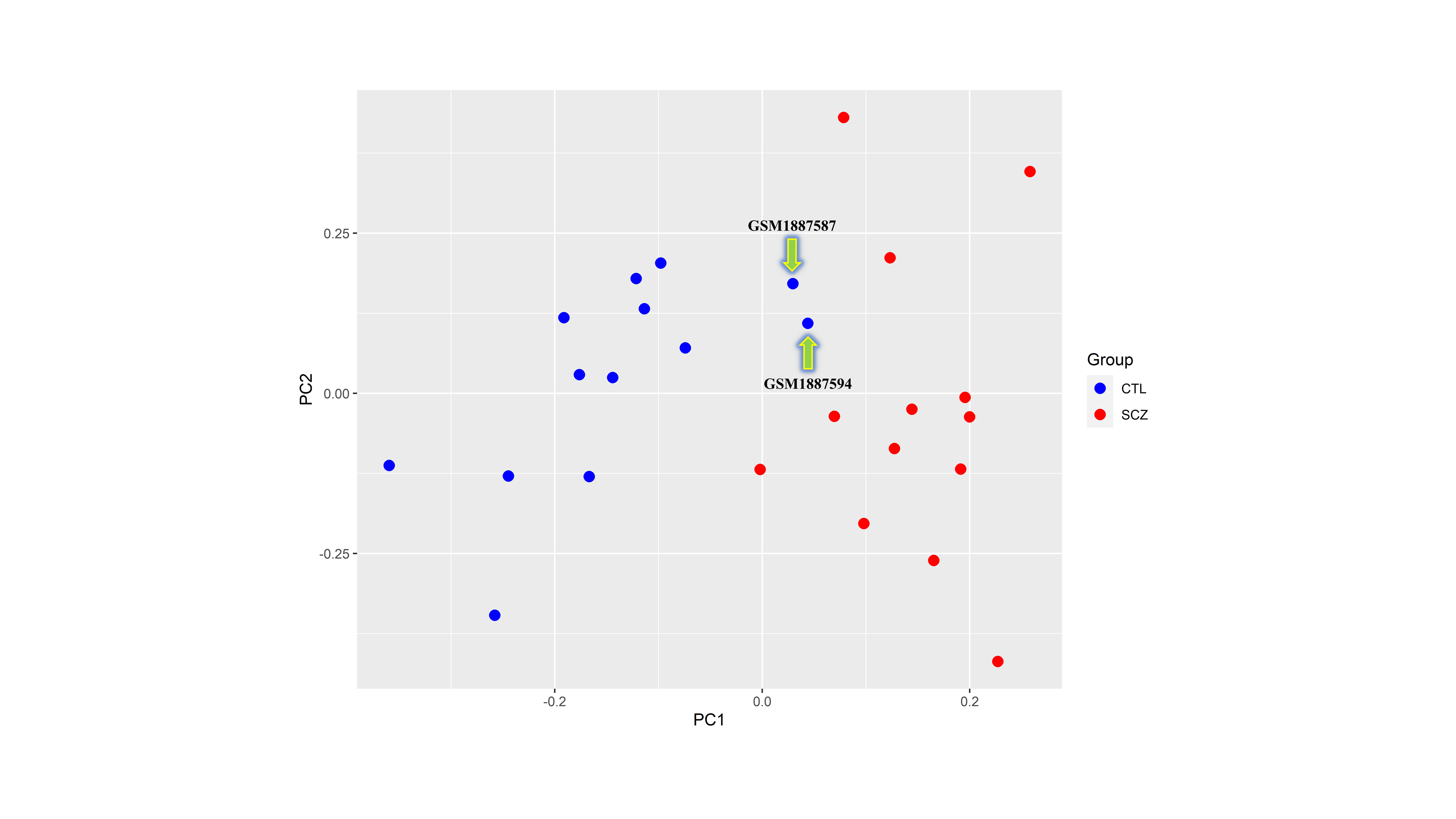
Figure S2.** Principal component analysis (PCA) for the GSE73129 dataset. All samples are segregated by condition group (on PC1). GSM1887587 and GSM1887594 (two control samples) were removed from further analysis in order to their wrong spatial enrichment. CTL, control; SCZ, schizophrenia.
